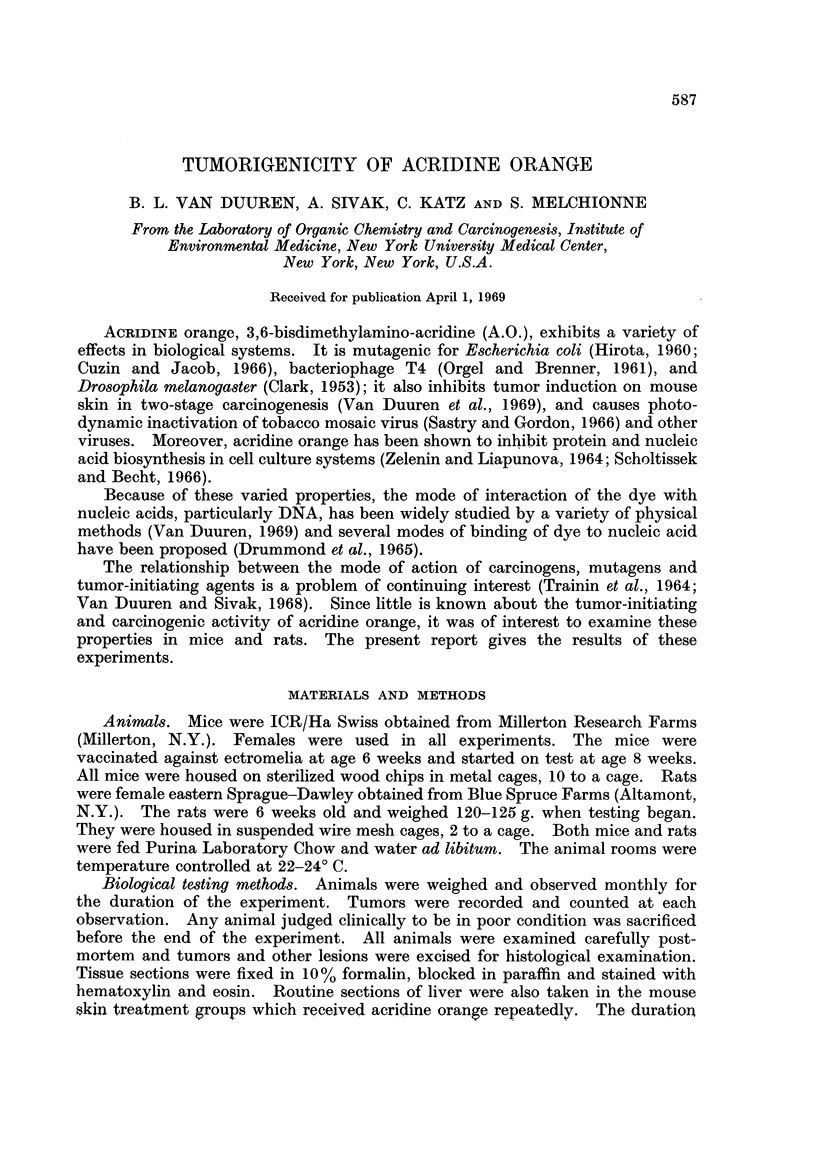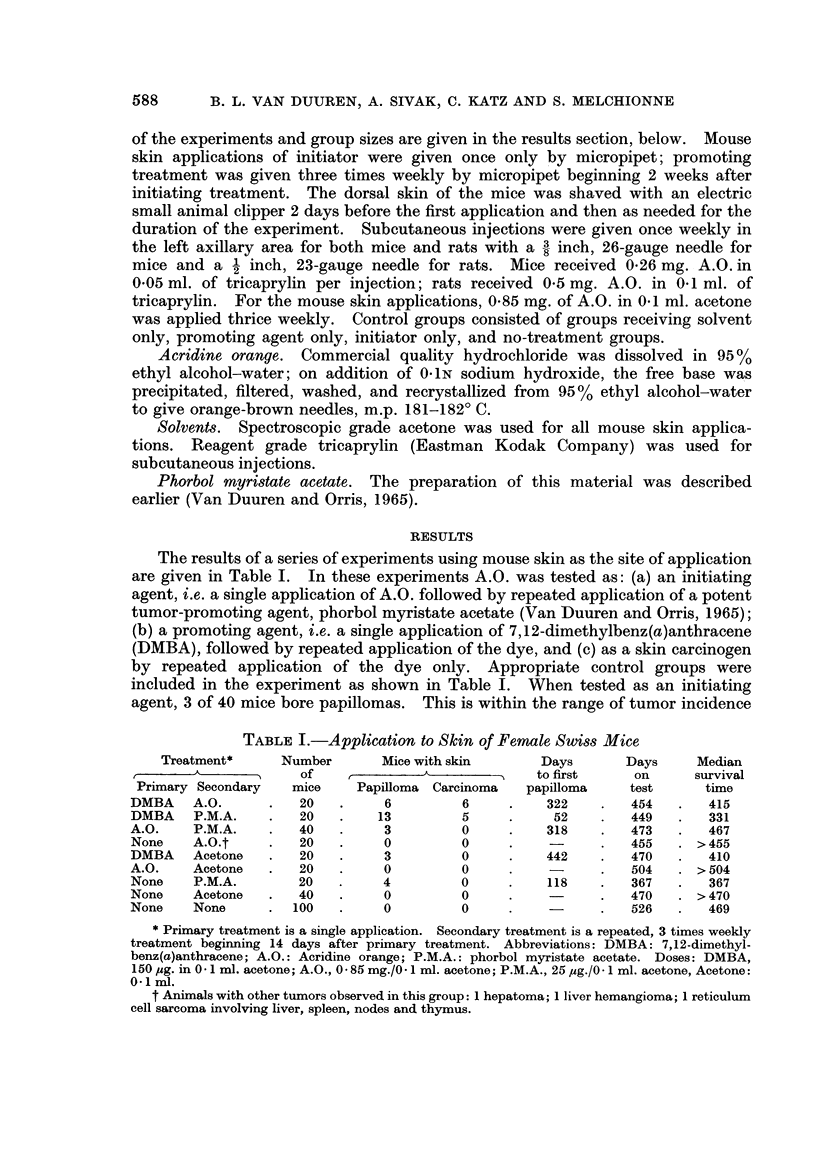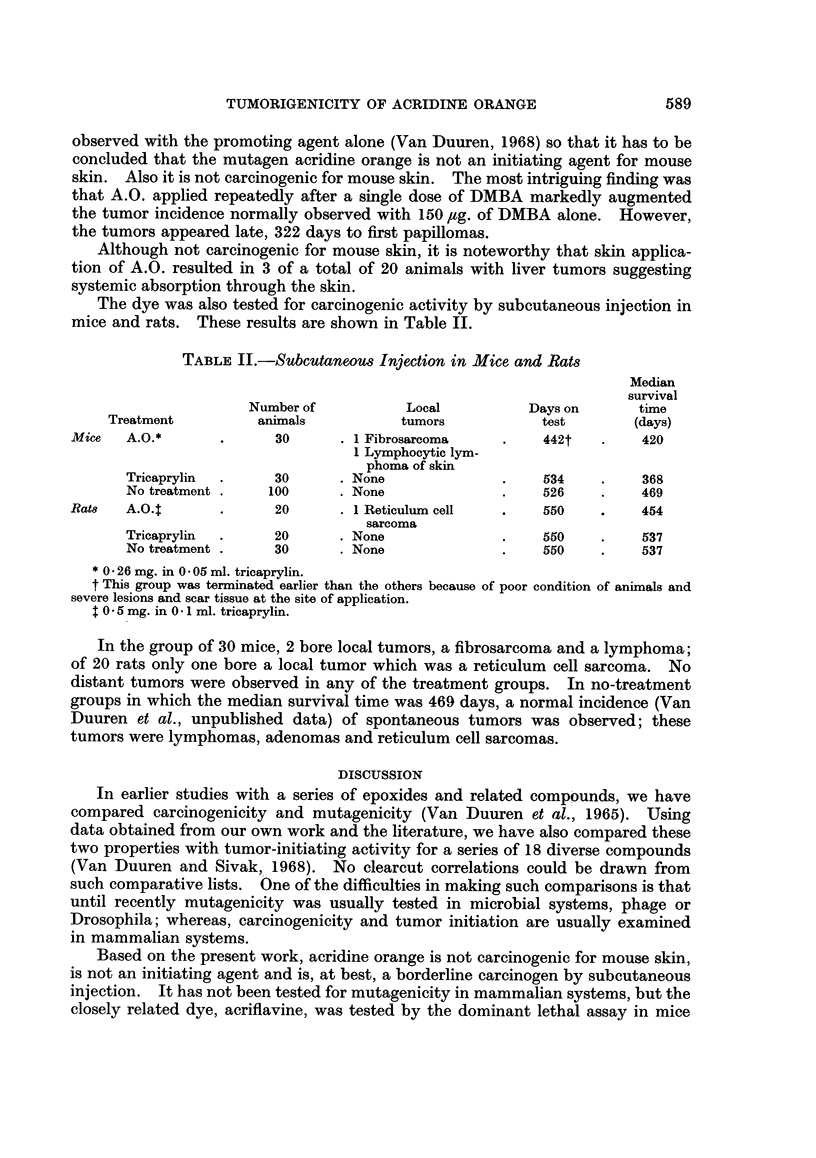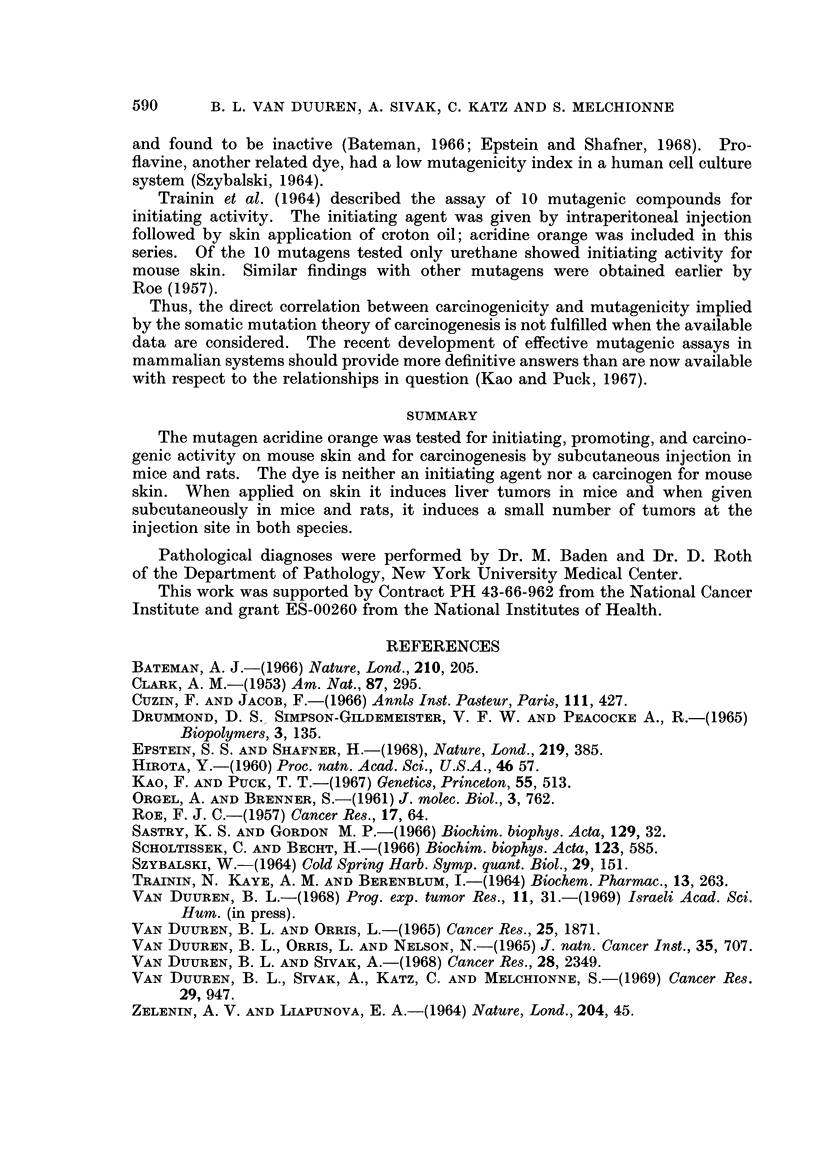# Tumorigenicity of acridine orange.

**DOI:** 10.1038/bjc.1969.72

**Published:** 1969-09

**Authors:** B. L. Van Duuren, A. Sivak, C. Katz, S. Melchionne


### TUMORIGENICITY OF ACRIDINE ORANGE

B. L. VAN DUUREN, A. SIVAK, C. KATZ AND S. MELCHIONNE

*From the Laboratory of Organic Chemistry and Carcinogenesis, Institute of Environmental Medicine, New York University Medical Center, New York, New York, U.S.A.*

Received for publication April 1, 1969

ACRIDINE orange, 3,6-bisdimethylamino-acridine (A.O.), exhibits a variety of effects in biological systems. It is mutagenic for *Escherichia coli* (Hirota, 1960; Cuzin and Jacob, 1966), bacteriophage T4 (Orgel and Brenner, 1961), and *Drosophila melanogaster* (Clark, 1953); it also inhibits tumor induction on mouse skin in two-stage carcinogenesis (Van Duuren *et al.*, 1969), and causes photodynamic inactivation of tobacco mosaic virus (Sastry and Gordon, 1966) and other viruses. Moreover, acridine orange has been shown to inhibit protein and nucleic acid biosynthesis in cell culture systems (Zelenin and Liapunova, 1964; Scholtissek and Becht, 1966).

Because of these varied properties, the mode of interaction of the dye with nucleic acids, particularly DNA, has been widely studied by a variety of physical methods (Van Duuren, 1969) and several modes of binding of dye to nucleic acid have been proposed (Drummond *et al.*, 1965).

The relationship between the mode of action of carcinogens, mutagens and tumor-initiating agents is a problem of continuing interest (Trainin *et al.*, 1964; Van Duuren and Sivak, 1968). Since little is known about the tumor-initiating and carcinogenic activity of acridine orange, it was of interest to examine these properties in mice and rats. The present report gives the results of these experiments.

### MATERIALS AND METHODS

*Animals.* Mice were ICR/Ha Swiss obtained from Millerton Research Farms (Millerton, N.Y.). Females were used in all experiments. The mice were vaccinated against ectromelia at age 6 weeks and started on test at age 8 weeks. All mice were housed on sterilized wood chips in metal cages, 10 to a cage. Rats were female eastern Sprague–Dawley obtained from Blue Spruce Farms (Altamont, N.Y.). The rats were 6 weeks old and weighed 120–125 g. when testing began. They were housed in suspended wire mesh cages, 2 to a cage. Both mice and rats were fed Purina Laboratory Chow and water *ad libitum.* The animal rooms were temperature controlled at 22–24° C.

*Biological testing methods.* Animals were weighed and observed monthly for the duration of the experiment. Tumors were recorded and counted at each observation. Any animal judged clinically to be in poor condition was sacrificed before the end of the experiment. All animals were examined carefully post-mortem and tumors and other lesions were excised for histological examination. Tissue sections were fixed in 10% formalin, blocked in paraffin and stained with hematoxylin and eosin. Routine sections of liver were also taken in the mouse skin treatment groups which received acridine orange repeatedly. The duration of the experiments and group sizes are given in the results section, below. Mouse skin applications of initiator were given once only by micropipet; promoting treatment was given three times weekly by micropipet beginning 2 weeks after initiating treatment. The dorsal skin of the mice was shaved with an electric small animal clipper 2 days before the first application and then as needed for the duration of the experiment. Subcutaneous injections were given once weekly in the left axillary area for both mice and rats with a $\frac{3}{8}$ inch, 26-gauge needle for mice and a $\frac{1}{2}$ inch, 23-gauge needle for rats. Mice received 0·26 mg. A.O. in 0·05 ml. of tricaprylin per injection; rats received 0·5 mg. A.O. in 0·1 ml. of tricaprylin. For the mouse skin applications, 0·85 mg. of A.O. in 0·1 ml. acetone was applied thrice weekly. Control groups consisted of groups receiving solvent only, promoting agent only, initiator only, and no-treatment groups.

*Acridine orange.* Commercial quality hydrochloride was dissolved in 95% ethyl alcohol–water; on addition of 0·1N sodium hydroxide, the free base was precipitated, filtered, washed, and recrystallized from 95% ethyl alcohol–water to give orange-brown needles, m.p. 181–182° C.

*Solvents.* Spectroscopic grade acetone was used for all mouse skin applications. Reagent grade tricaprylin (Eastman Kodak Company) was used for subcutaneous injections.

*Phorbol myristate acetate.* The preparation of this material was described earlier (Van Duuren and Orris, 1965).

### RESULTS

The results of a series of experiments using mouse skin as the site of application are given in Table I. In these experiments A.O. was tested as: (a) an initiating agent, *i.e.* a single application of A.O. followed by repeated application of a potent tumor-promoting agent, phorbol myristate acetate (Van Duuren and Orris, 1965); (b) a promoting agent, *i.e.* a single application of 7,12-dimethylbenz(*a*)anthracene (DMBA), followed by repeated application of the dye, and (c) as a skin carcinogen by repeated application of the dye only. Appropriate control groups were included in the experiment as shown in Table I. When tested as an initiating agent, 3 of 40 mice bore papillomas. This is within the range of tumor incidence

TABLE I.—*Application to Skin of Female Swiss Mice*

| Treatment* | | Number of mice | Mice with skin | | Days to first papilloma | Days on test | Median survival time |
|---|---|---|---|---|---|---|---|
| Primary | Secondary | | Papilloma | Carcinoma | | | |
| DMBA | A.O. | 20 | 6 | 6 | 322 | 454 | 415 |
| DMBA | P.M.A. | 20 | 13 | 5 | 52 | 449 | 331 |
| A.O. | P.M.A. | 40 | 3 | 0 | 318 | 473 | 467 |
| None | A.O.† | 20 | 0 | 0 | — | 455 | >455 |
| DMBA | Acetone | 20 | 3 | 0 | 442 | 470 | 410 |
| A.O. | Acetone | 20 | 0 | 0 | — | 504 | >504 |
| None | P.M.A. | 20 | 4 | 0 | 118 | 367 | 367 |
| None | Acetone | 40 | 0 | 0 | — | 470 | >470 |
| None | None | 100 | 0 | 0 | — | 526 | 469 |

* Primary treatment is a single application. Secondary treatment is a repeated, 3 times weekly treatment beginning 14 days after primary treatment. Abbreviations: DMBA: 7,12-dimethylbenz(*a*)anthracene; A.O.: Acridine orange; P.M.A.: phorbol myristate acetate. Doses: DMBA, 150 μg. in 0·1 ml. acetone; A.O., 0·85 mg./0·1 ml. acetone; P.M.A., 25 μg./0·1 ml. acetone, Acetone: 0·1 ml.

† Animals with other tumors observed in this group: 1 hepatoma; 1 liver hemangioma; 1 reticulum cell sarcoma involving liver, spleen, nodes and thymus.

observed with the promoting agent alone (Van Duuren, 1968) so that it has to be concluded that the mutagen acridine orange is not an initiating agent for mouse skin. Also it is not carcinogenic for mouse skin. The most intriguing finding was that A.O. applied repeatedly after a single dose of DMBA markedly augmented the tumor incidence normally observed with 150 $\mu$g. of DMBA alone. However, the tumors appeared late, 322 days to first papillomas.

Although not carcinogenic for mouse skin, it is noteworthy that skin application of A.O. resulted in 3 of a total of 20 animals with liver tumors suggesting systemic absorption through the skin.

The dye was also tested for carcinogenic activity by subcutaneous injection in mice and rats. These results are shown in Table II.

TABLE II.—*Subcutaneous Injection in Mice and Rats*

| | Treatment | Number of animals | Local tumors | Days on test | Median survival time (days) |
|---|---|---|---|---|---|
| *Mice* | A.O.* | 30 | 1 Fibrosarcoma<br>1 Lymphocytic lymphoma of skin | 442† | 420 |
| | Tricaprylin | 30 | None | 534 | 368 |
| | No treatment | 100 | None | 526 | 469 |
| *Rats* | A.O.‡ | 20 | 1 Reticulum cell sarcoma | 550 | 454 |
| | Tricaprylin | 20 | None | 550 | 537 |
| | No treatment | 30 | None | 550 | 537 |

* 0·26 mg. in 0·05 ml. tricaprylin.

† This group was terminated earlier than the others because of poor condition of animals and severe lesions and scar tissue at the site of application.

‡ 0·5 mg. in 0·1 ml. tricaprylin.

In the group of 30 mice, 2 bore local tumors, a fibrosarcoma and a lymphoma; of 20 rats only one bore a local tumor which was a reticulum cell sarcoma. No distant tumors were observed in any of the treatment groups. In no-treatment groups in which the median survival time was 469 days, a normal incidence (Van Duuren *et al.*, unpublished data) of spontaneous tumors was observed; these tumors were lymphomas, adenomas and reticulum cell sarcomas.

### DISCUSSION

In earlier studies with a series of epoxides and related compounds, we have compared carcinogenicity and mutagenicity (Van Duuren *et al.*, 1965). Using data obtained from our own work and the literature, we have also compared these two properties with tumor-initiating activity for a series of 18 diverse compounds (Van Duuren and Sivak, 1968). No clearcut correlations could be drawn from such comparative lists. One of the difficulties in making such comparisons is that until recently mutagenicity was usually tested in microbial systems, phage or Drosophila; whereas, carcinogenicity and tumor initiation are usually examined in mammalian systems.

Based on the present work, acridine orange is not carcinogenic for mouse skin, is not an initiating agent and is, at best, a borderline carcinogen by subcutaneous injection. It has not been tested for mutagenicity in mammalian systems, but the closely related dye, acriflavine, was tested by the dominant lethal assay in mice and found to be inactive (Bateman, 1966; Epstein and Shafner, 1968). Proflavine, another related dye, had a low mutagenicity index in a human cell culture system (Szybalski, 1964).

Trainin *et al.* (1964) described the assay of 10 mutagenic compounds for initiating activity. The initiating agent was given by intraperitoneal injection followed by skin application of croton oil; acridine orange was included in this series. Of the 10 mutagens tested only urethane showed initiating activity for mouse skin. Similar findings with other mutagens were obtained earlier by Roe (1957).

Thus, the direct correlation between carcinogenicity and mutagenicity implied by the somatic mutation theory of carcinogenesis is not fulfilled when the available data are considered. The recent development of effective mutagenic assays in mammalian systems should provide more definitive answers than are now available with respect to the relationships in question (Kao and Puck, 1967).

SUMMARY

The mutagen acridine orange was tested for initiating, promoting, and carcinogenic activity on mouse skin and for carcinogenesis by subcutaneous injection in mice and rats. The dye is neither an initiating agent nor a carcinogen for mouse skin. When applied on skin it induces liver tumors in mice and when given subcutaneously in mice and rats, it induces a small number of tumors at the injection site in both species.

Pathological diagnoses were performed by Dr. M. Baden and Dr. D. Roth of the Department of Pathology, New York University Medical Center.

This work was supported by Contract PH 43-66-962 from the National Cancer Institute and grant ES-00260 from the National Institutes of Health.

### REFERENCES

BATEMAN, A. J.—(1966) *Nature, Lond.*, **210**, 205.
CLARK, A. M.—(1953) *Am. Nat.*, **87**, 295.
CUZIN, F. AND JACOB, F.—(1966) *Annls Inst. Pasteur, Paris*, **111**, 427.
DRUMMOND, D. S. SIMPSON-GILDEMEISTER, V. F. W. AND PEACOCKE A., R.—(1965) *Biopolymers*, **3**, 135.
EPSTEIN, S. S. AND SHAFNER, H.—(1968), *Nature, Lond.*, **219**, 385.
HIROTA, Y.—(1960) *Proc. natn. Acad. Sci., U.S.A.*, **46** 57.
KAO, F. AND PUCK, T. T.—(1967) *Genetics, Princeton*, **55**, 513.
ORGEL, A. AND BRENNER, S.—(1961) *J. molec. Biol.*, **3**, 762.
ROE, F. J. C.—(1957) *Cancer Res.*, **17**, 64.
SASTRY, K. S. AND GORDON M. P.—(1966) *Biochim. biophys. Acta*, **129**, 32.
SCHOLTISSEK, C. AND BECHT, H.—(1966) *Biochim. biophys. Acta*, **123**, 585.
SZYBALSKI, W.—(1964) *Cold Spring Harb. Symp. quant. Biol.*, **29**, 151.
TRAININ, N. KAYE, A. M. AND BERENBLUM, I.—(1964) *Biochem. Pharmac.*, **13**, 263.
VAN DUUREN, B. L.—(1968) *Prog. exp. tumor Res.*, **11**, 31.—(1969) *Israeli Acad. Sci. Hum.* (in press).
VAN DUUREN, B. L. AND ORRIS, L.—(1965) *Cancer Res.*, **25**, 1871.
VAN DUUREN, B. L., ORRIS, L. AND NELSON, N.—(1965) *J. natn. Cancer Inst.*, **35**, 707.
VAN DUUREN, B. L. AND SIVAK, A.—(1968) *Cancer Res.*, **28**, 2349.
VAN DUUREN, B. L., SIVAK, A., KATZ, C. AND MELCHIONNE, S.—(1969) *Cancer Res.* **29**, 947.
ZELENIN, A. V. AND LIAPUNOVA, E. A.—(1964) *Nature, Lond.*, **204**, 45.